# Gut microbiota-derived short-chain fatty acids attenuate placental ferroptosis and insulin resistance in gestational diabetes via the ACSL4/LPCAT3 pathway

**DOI:** 10.3389/fmicb.2026.1715392

**Published:** 2026-05-20

**Authors:** Yang Han, Lingyi Kong, Tao Wang, Niankun Chen, Dongyu Wang, Zilian Wang, Yanxin Wu

**Affiliations:** 1 Department of Obstetrics, The First Affiliated Hospital, Sun Yat-sen University, Guangzhou, Guangdong, China; 2 Research and Development Department, Klarity Medical & Equipment (GZ) Co., Ltd., Guangzhou, Guangdong, China

**Keywords:** ferroptosis, gestational diabetes mellitus, gut microbiota, insulin resistance, short-chain fatty acids

## Abstract

**Objective:**

Gestational diabetes mellitus (GDM) is associated with gut microbiota dysbiosis and placental dysfunction. This study aimed to investigate whether gut microbiota-derived short-chain fatty acids (SCFAs) ameliorate placental injury and insulin resistance in GDM by suppressing ferroptosis, a novel form of iron-dependent cell death.

**Methods:**

A GDM rat model was induced by streptozotocin, and gut microbiota was depleted via a broad-spectrum antibiotic cocktail. Physiological parameters, gut microbiota composition, and SCFA levels were assessed. Placental and metabolic tissues were analyzed for ferroptosis markers (Fe^2+^, MDA, ROS), key regulatory enzymes (GPX4, ACSL4, LPCAT3), polyunsaturated fatty acid (PUFA) profiles, and inflammation. An *in vitro* insulin resistance model in human trophoblast cells (HTR-8/SVneo) was used to validate the causal role of SCFAs.

**Results:**

GDM and antibiotic-induced dysbiosis led to a significant depletion of SCFA-producing bacteria and reduced colonic SCFA levels. This was concomitant with severe placental ferroptosis, characterized by iron accumulation, lipid peroxidation, and downregulation of GPX4 alongside upregulation of ACSL4 and LPCAT3. PUFA substrates were significantly consumed, and systemic inflammation was elevated. *In vitro*, insulin resistance induced trophoblast ferroptosis and dysfunction, which was effectively rescued by SCFA treatment. SCFAs restored GPX4 expression, suppressed ACSL4/LPCAT3, preserved PUFAs, and attenuated inflammation.

**Conclusion:**

Gut microbiota-derived SCFAs play a critical protective role in GDM by inhibiting the ACSL4/LPCAT3-mediated ferroptotic pathway in placental trophoblasts, thereby improving insulin sensitivity and mitigating cellular injury. Our findings highlight the gut-placenta axis as a potential therapeutic target for GDM, warranting further validation in human studies.

## 1 Introduction

Gestational diabetes mellitus (GDM) is one of the most common complications during pregnancy, around 14% of pregnancies globally are affected by gestational diabetes, characterized by the onset or first recognition of glucose intolerance resulting in hyperglycemia (Sweeting et al., 2024). GDM is associated with metabolic disturbances such as obesity, low-grade inflammation, and insulin resistance, and contributes to a high incidence of adverse pregnancy outcomes (Zehravi et al., 2021). Identifying strategies to reduce the incidence of GDM and improve clinical prognoses remains an important research focus.

Ferroptosis is a novel form of regulated cell death driven by iron dependency and characterized by dysregulated iron metabolism, amino acid metabolism, and lipid peroxidation, leading to iron accumulation, elevated reactive oxygen species (ROS), and oxidative damage (Shintoku et al., 2017; Yang et al., 2016). Glutathione peroxidase 4 (GPX4), the only enzyme capable of reducing lipid hydroperoxides within cells, plays a central role in protecting against membrane lipid peroxidation. Its deficiency results in increased ROS and activation of lipoxygenases (LOXs), promoting peroxidation of membrane polyunsaturated fatty acids (PUFAs) and ultimately leading to ferroptosis (Xie et al., 2023).

Acyl-CoA synthetase long-chain family member 4 (ACSL4) and lysophosphatidylcholine acyltransferase 3 (LPCAT3) are two key enzymes involved in esterifying PUFAs into membrane phospholipids and facilitating lipid peroxidation (Gupta et al., 2023; Sha et al., 2021). ACSL4 catalyzes the conversion of arachidonic acid (AA) and adrenic acid (AdA) into their CoA derivatives, which are then incorporated into phospholipids by LPCAT3. These lipid substrates are subsequently oxidized by LOXs, resulting in membrane damage and ferroptosis. Thus, suppression of ACSL4 and LPCAT3 may reduce lipid peroxide accumulation and inhibit ferroptosis. Growing evidence suggests that ferroptosis is implicated in the pathophysiology of various pregnancy-related disorders, including preeclampsia, GDM, and miscarriage (Zhao et al., 2023; You et al., 2024); however, the underlying mechanisms remain incompletely understood.

The gut microbiota plays a pivotal role in regulating host metabolism and immune responses, thereby influencing disease progression. Recent studies have highlighted the ability of gut microbes and their metabolites, particularly short-chain fatty acids (SCFAs), to modulate ferroptosis. SCFAs enhance mitochondrial oxidative phosphorylation and *β*-oxidation under physiological conditions, reduce ROS production, and promote anti-inflammatory and antioxidant responses (Rose et al., 2018; Li X. et al., 2022). Moreover, SCFAs upregulate key anti-ferroptotic proteins such as GPX4 and hepcidin via the Nrf2/GPX4 signaling pathway, thereby suppressing ferroptosis in colitis models (Shanmugam et al., 2014; Chen et al., 2024). SCFAs also support mitochondrial biogenesis and function through direct or indirect regulation of the PPARγ/PGC1α axis (He et al., 2024; den Besten et al., 2015). Collectively, these findings suggest that SCFAs can modulate cellular ferroptosis and influence disease development (Chen et al., 2024; Wang et al., 2025). Nevertheless, the role of SCFAs in regulating trophoblast ferroptosis and insulin resistance in GDM remains unclear.

In this study, we employed both *in vivo* and *in vitro* models to investigate whether gut microbiota-derived SCFAs ameliorate insulin resistance and placental ferroptosis in GDM by modulating the ACSL4/LPCAT3 pathway. We integrated multi-omics analyses, including 16S rRNA sequencing and targeted metabolomics analysis using GC–MS, to identify key microbial and metabolic features associated with GDM. Furthermore, we validated the functional role of SCFAs in regulating ferroptosis and inflammatory responses in a trophoblast insulin resistance model. Our findings provide new insights into the gut microbiota-SCFA-ferroptosis axis and its potential implications for GDM pathogenesis and therapy.

## 2 Materials and methods

### 2.1 Animal study

A total of 36 pregnant Wistar rats (weight 200 ± 20 g) were supplied by Shanghai Bikai Keyi Biotechnology Co., Ltd. (License No. SCXK(Hu)2023–0009). Animals were housed under specific pathogen-free (SPF) conditions at the animal facility (License No. SYXK(Su)2021–0007). The housing environment was maintained at 22 ± 2 °C, with a 12-h light/dark cycle. Rats had ad libitum access to standard chow and water throughout the study.

The gestational diabetes mellitus (GDM) model was induced on gestational day (GD) 0 by a single intraperitoneal injection of streptozotocin (STZ; MedChemExpress, #HY-13753, Shanghai, China) at a dose of 40 mg/kg dissolved in 0.1 M citrate buffer (pH 4.5) after overnight fasting (Takahashi et al., 2024). Control rats received citrate buffer only. Blood glucose levels were measured 72 h post-injection using a glucometer. Rats with fasting blood glucose ≥ 16.7 mmol/L were considered GDM and included in the study.

Rats were randomly assigned to three groups (*n* = 12/group): NC group: Normal control rats, injected with citrate buffer. Model group: GDM rats induced by STZ. Abx group: GDM rats treated with a broad-spectrum antibiotic cocktail in drinking water from GD1 until the end of the experiment. The broad-spectrum antibiotic cocktail consisted of four antibiotics targeting distinct bacterial populations: ampicillin (MedChemExpress, #HY-B0522, 1 g/L), a *β*-lactam antibiotic inhibiting cell wall synthesis in Gram-positive and some Gram-negative bacteria; neomycin sulfate (MedChemExpress, #HY-B0470, 1 g/L), an aminoglycoside inhibiting protein synthesis primarily in Gram-negative bacteria; metronidazole (Solarbio, #M8060, Beijing, China, 1 g/L), a nitroimidazole effective against anaerobic bacteria and protozoa; and vancomycin (MedChemExpress, #HY-B0671, 0.5 g/L), a glycopeptide antibiotic specifically targeting Gram-positive bacteria by inhibiting cell wall synthesis (Reikvam et al., 2011). This combination was selected to achieve comprehensive depletion of the gut microbiota, including aerobic, anaerobic, Gram-positive, and Gram-negative bacteria.

**Figure 1:**
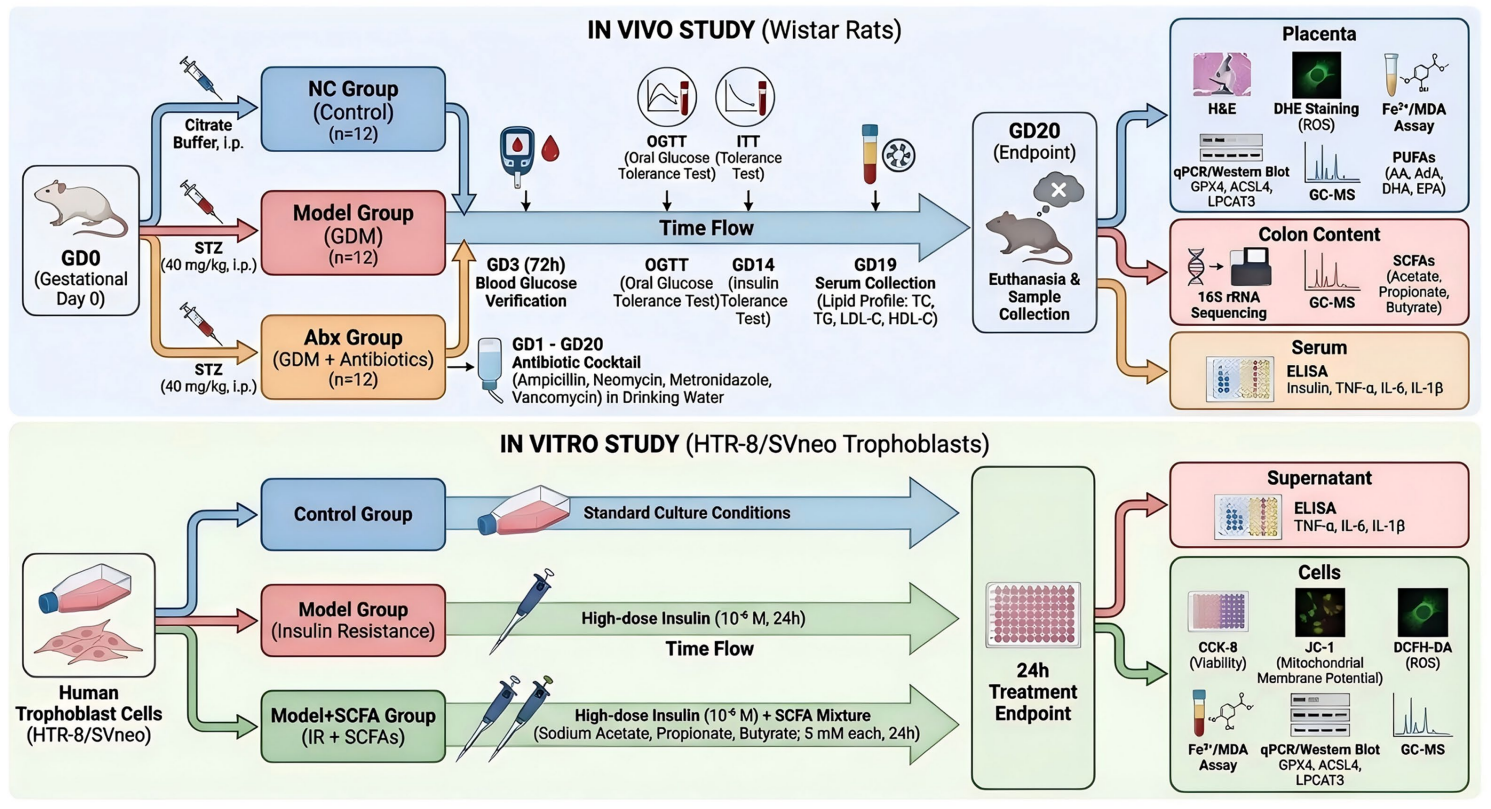
Schematic of the proposed mechanism by which the gut microbiota-placenta axis regulates trophoblast ferroptosis in gestational diabetes mellitus (GDM). During a healthy pregnancy (left panel), a normobiotic gut microbiota produces abundant short-chain fatty acids (SCFAs). These microbial metabolites suppress ferroptosis in placental trophoblasts by upregulating the key antioxidant enzyme glutathione peroxidase 4 (GPX4) and downregulating the pro-ferroptotic enzymes Acyl-CoA synthetase long-chain family member 4 (ACSL4) and lysophosphatidylcholine acyltransferase 3 (LPCAT3). This action preserves placental health and supports a normal pregnancy outcome. Conversely, in the GDM state (right panel), gut microbiota dysbiosis leads to a significant deficiency in SCFAs. The lack of SCFAs results in the disinhibition of ferroptosis in trophoblasts, characterized by the downregulation of GPX4 and upregulation of ACSL4 and LPCAT3. This molecular shift facilitates the peroxidation of polyunsaturated fatty acids (PUFAs), culminating in trophoblast ferroptosis, placental injury, inflammation, and contributing to the pathophysiology of GDM.

The rationale for including the Abx group was to specifically investigate the contribution of gut microbiota depletion and the consequent reduction of SCFAs to the pathogenesis of placental ferroptosis and metabolic dysfunction in the context of GDM. In this group, GDM was first induced by STZ, followed by the administration of the broad-spectrum antibiotic cocktail from gestational day 1 onwards. This sequential design (STZ induction first, then antibiotic treatment) allowed us to model a common clinical scenario where GDM is associated with, and potentially exacerbated by, pre-existing or concomitant gut dysbiosis. By comparing the Model (GDM only) and Abx (GDM + microbiota depletion) groups, we aimed to dissect the specific role of the gut microbiota and its metabolic output (SCFAs) in modulating the severity of GDM-induced placental injury, beyond the effect of hyperglycemia and insulin resistance alone (Figure 1).

Body weight, oral glucose tolerance test (OGTT), and insulin tolerance test (ITT) were performed on GD14. Blood samples were collected for measurement of total cholesterol (TC), triglycerides (TG), low-density lipoprotein cholesterol (LDL-C), and high-density lipoprotein cholesterol (HDL-C) levels.

On GD20, rats were euthanized under deep anesthesia with pentobarbital sodium (50 mg/kg, i.p.). Colon contents were collected for 16S rRNA sequencing and short-chain fatty acid (SCFA) analysis via GC–MS. Placental and pancreatic tissues were harvested for subsequent molecular and histological analyses. SCFA analysis was performed on colonic contents from three randomly selected rats per experimental group (*n* = 3), representing a subset of the 12 animals per group used for the main study. Sample size was determined *a priori* using G*Power software (version 3.1.9.7). Based on preliminary experiments evaluating the primary outcome variable, placental malondialdehyde (MDA) levels, we assumed a large effect size (Cohen’s d = 1.2) for the overall difference among the NC, Model, and Abx groups. With a significance level (*α*) of 0.05 and a desired statistical power (1 − *β*) of 0.80, the calculated sample size was 6 animals per group. Considering the potential for pregnancy-related loss, variability in the GDM model, and the need to ensure sufficient biological material for multiple downstream analyses, we ultimately included 12 animals in each group.

All animal procedures were conducted in accordance with the NIH Guide for the Care and Use of Laboratory Animals and were approved by the Animal Ethics Committee of Clinical Research and Animal Trials of the First Affiliated Hospital of Sun Yat-sen University (Approval No. LunShenDong[2025]132).

### 2.2 Cell culture

The human placental trophoblast cell line HTR-8/SVneo was obtained from the American Type Culture Collection (ATCC, Manassas, VA, USA). Cells were cultured in RPMI 1640 medium (GIBCO, #A33823, NY, USA) supplemented with 10% fetal bovine serum (FBS; Hyclone, #SH30070.03, Logan, UT, USA) and 1% penicillin–streptomycin (Thermo Fisher Scientific, #15140148, Pittsburgh, PA, USA). Cultures were maintained at 37 °C in a humidified atmosphere containing 5% CO_2_.

An insulin resistance (IR) model in HTR-8/SVneo cells was established using a previously described method with modifications (Li L. et al., 2022). Briefly, near-confluent cells were cultured in serum-free RPMI 1640 medium for 12 h to synchronize the cell cycle. Subsequently, the medium was replaced with complete growth medium containing a high concentration of insulin (MedChemExpress, #HY-P0035, Shanghai, China) at 10^−6^ M and incubated for 24 h to induce insulin resistance (Li L. et al., 2022).

Experimental groups and treatments were designed as follows: Control group: Cells cultured under standard conditions without any treatment. Model group: Cells subjected to the insulin resistance induction protocol. Model+SCFA group: following the establishment of the insulin resistance model, cells were treated with a mixture of short-chain fatty acids (SCFAs) including sodium acetate (Sigma-Aldrich, #71251, St. Louis, MO, USA), sodium propionate (Sigma-Aldrich, #8.00605), and sodium butyrate (Sigma-Aldrich, #W222100). The SCFA mixture was dissolved directly into the cell culture medium at a final concentration of 5 mM for each SCFA. The treatment was applied concurrently with the insulin challenge and maintained for 24 h (Otten et al., 2023).

After the respective treatments, cell culture supernatants were collected, centrifuged at 1000 × g for 10 min to remove cellular debris, and aliquoted for subsequent analysis of inflammatory cytokines. Adherent cells were washed twice with cold phosphate-buffered saline (PBS). For protein extraction, cells were lysed directly on the plate using RIPA lysis buffer (KeyGEN BioTECH, #KGB5203, Nanjing, China) supplemented with 1 mM PMSF (Amresco, #97064–898, OH, USA). The lysates were scraped, transferred to microcentrifuge tubes, and centrifuged at 12,000 × g for 15 min at 4 °C to obtain clear supernatants for Western blot analysis. For RNA extraction, cells were lysed with TRIzol reagent (Takara, #9109, Japan) and processed according to the manufacturer’s instructions. For assessment of intracellular metabolites (Fe^2+^, MDA, ROS) and mitochondrial function, cells were harvested by trypsinization (GIBCO, #15090046), counted, and prepared as single-cell suspensions for immediate analysis or appropriately stored until assayed.

### 2.3 Oral glucose tolerance test (OGTT)

The oral glucose tolerance test (OGTT) was performed on gestational day 14 to assess glucose homeostasis in the pregnant rats. After a 12-h overnight fast (with free access to water), rats in all groups received an oral gavage of a glucose solution (2.0 g glucose per kg body weight, prepared as a 50% w/v solution in sterile water). Blood samples were collected from the tail vein immediately before (0 min) and at 30, 60, 90, and 120 min after glucose administration. Blood glucose levels were measured using a portable glucometer (Accu-Chek Performa, Roche Diagnostics GmbH, Germany). The area under the curve (AUC) for the OGTT was calculated using the trapezoidal rule to provide a quantitative assessment of glucose tolerance.

### 2.4 Serum lipid profile analysis

On gestational day 19, rats were euthanized, and blood samples were collected from the abdominal aorta. The blood samples were allowed to clot at room temperature for 2 h and then centrifuged at 3500 rpm (approximately 1,500 × g) for 10 min to obtain serum. The serum levels of total cholesterol (TC), triglycerides (TG), low-density lipoprotein cholesterol (LDL-C), and high-density lipoprotein cholesterol (HDL-C) were analyzed using a fully automatic biochemical analyzer (PF-300VET, Shenzhen Pukang Electronic Co., Ltd., China).

### 2.5 Serum insulin and inflammatory cytokine measurement by ELISA

Serum levels of insulin in rats were quantified using a commercial Rat Insulin ELISA Kit (Solarbio, #SEKR-0033, Beijing, China). Serum and cell culture supernatant levels of TNF-*α*, IL-6, and IL-1β were measured using specific ELISA kits (R&D Systems: Rat TNF-α, #RTA00; Human TNF-α, #QK210; Human IL-6, #D6050B. MULTI SCIENCES: Rat IL-6, #EK306; Rat IL-1β, #EK301B; Human IL-1β, #EK101B; Zhejiang, China). All assays were strictly performed according to the manufacturers’ protocols.

Briefly, for serum preparation, blood samples were collected and centrifuged at 3000 × g for 15 min at 4 °C. Cell culture supernatants were centrifuged at 1000 × g for 10 min to remove debris. All samples were stored at −80 °C until analysis. Standard solutions were prepared by serial dilution as instructed. 100 μL of standards, blanks, or samples were added to the appropriate wells of the pre-coated plate and incubated. After incubation and a washing step (using the provided wash buffer), a biotinylated detection antibody was added, followed by another incubation and wash. Subsequently, streptavidin-HRP was added. After a final wash, tetramethylbenzidine (TMB) substrate was added for color development. The reaction was stopped with a stop solution, and the absorbance was immediately measured at 450 nm (with wavelength correction set to 540 nm or 570 nm) using a full-wavelength microplate analyzer (HBS-SCANX, Nanjing Detie Biotechnology Co., Ltd., China). The concentration of each analyte was calculated by interpolating from the respective standard curve.

### 2.6 Measurement of Fe^2+^ and malondialdehyde (MDA) levels

Intracellular and placental tissue Fe^2+^ levels were measured using an Iron Assay Kit (Colorimetric) (Solarbio, #BC5415, Beijing, China). Approximately 0.1 g of placental tissue was homogenized in 1 mL of the provided extraction buffer on ice. Cell pellets were resuspended and sonicated in extraction buffer. The homogenates were centrifuged at 10,000 × *g* for 10 min at 4 °C to obtain the supernatant. A series of Fe^2+^ standards (0.78125 to 100 μmol/L) were prepared from the provided stock solution. 200 μL of sample, standard, or blank (distilled water) was mixed with 100 μL of reagent in a microcentrifuge tube. After incubation at 37 °C for 10 min, 100 μL of chloroform was added. The mixture was vortexed vigorously for 5 min and then centrifuged at 12,000 × *g* for 10 min at room temperature. 200 μL of the upper aqueous phase was carefully transferred to a 96-well plate, and the absorbance was measured at 593 nm using the microplate analyzer (HBS-SCANX). The Fe^2+^ concentration was determined from the standard curve and normalized to the sample protein concentration (μmol/mg prot) measured by a BCA Protein Assay Kit (KeyGEN BioTECH, #KGB2101, Nanjing, China).

The extent of lipid peroxidation was assessed by measuring malondialdehyde (MDA) levels using an MDA Content Assay Kit (Solarbio, #BC0025, Beijing, China). Placental tissue (0.1 g) or cell pellets were homogenized in 1 mL of the provided lysis buffer on ice. The homogenates were centrifuged at 10,000 × *g* for 10 min at 4 °C. 100 μL of the supernatant was mixed with 300 μL of the MDA working solution and 100 μL of reagent 3 in a microcentrifuge tube. The mixture was incubated in a 100 °C-water bath for 60 min, cooled on ice, and then centrifuged at 10,000 × *g* for 10 min at room temperature. 200 μL of the supernatant was transferred to a 96-well plate. The absorbance was measured at 532 nm and 600 nm for baseline correction. The MDA concentration was calculated according to the kit’s instructions using the formula provided and normalized to the sample protein concentration (nmol/mg prot).

### 2.7 Hematoxylin and eosin (H&E) staining

Placental tissue morphology was evaluated using standard hematoxylin and eosin (H&E) staining. Paraffin-embedded sections were baked at 60 °C for 3 h, deparaffinized in xylene, and rehydrated through a graded ethanol series. Sections were stained with hematoxylin for 2 min, differentiated in acid alcohol, and blued in water. Subsequent eosin staining was performed for 1 min. After dehydration through graded alcohols and xylene clearance, sections were mounted with neutral balsam. Histological images were acquired using a light microscope (DM4B, Leica Microsystems, Germany) under 40 × and 200 × magnification for morphological assessment.

### 2.8 Detection of reactive oxygen species in placental tissue

Reactive oxygen species (ROS) levels in placental tissues were assessed using dihydroethidium (DHE, Thermo Fisher Scientific, #D1168) fluorescence staining. Fresh-frozen or paraffin-embedded placental sections were brought to room temperature for 30 min. After washing with PBS, sections were incubated with 2 μmol/L DHE working solution (prepared in DMSO) at 37 °C for 30 min in the dark. Sections were then rinsed with PBS to remove unbound dye and counterstained with DAPI (Thermo Fisher Scientific, #D1306) for nuclear visualization. After mounting with antifade medium, images were captured under a fluorescence microscope (DM4B, Leica Microsystems, Germany) at 400 × magnification.

### 2.9 Quantitative PCR (qPCR)

Total RNA was extracted from tissues or cells using TRIzol reagent (Takara, #9109, Japan) according to the manufacturer’s instructions. RNA purity and concentration were determined using a NanoDrop 2000 spectrophotometer (Thermo Fisher Scientific, USA), with all samples having an A260/A280 ratio between 1.8 and 2.0. cDNA was synthesized from 500 ng of total RNA using the iScript cDNA Synthesis Kit (Bio-Rad, #1708891EDU, Hercules, CA, USA). Quantitative real-time PCR was performed using TB Green Premix Ex Taq (Tli RNase H Plus) (Takara, #RR420A, Japan) on an ABI 7500 Real-Time PCR System (Applied Biosystems, USA). The PCR cycling conditions were as follows: initial denaturation at 95 °C for 30 s, followed by 40 cycles of 95 °C for 5 s and 60 °C for 30 s. Melt curve analysis was performed to confirm the specificity of amplification. The sequences of all gene-specific primers used are listed in Table 1. The relative mRNA expression levels were calculated using the 2^−ΔΔCt^ method and normalized to the expression of *β*-actin.

**Table 1:** Primers used in quantitative real-time PCR.

Primers	Sequence (5′ → 3′)
GPX4 (Human)	Forward: TCACCAAGTTTGGACACCGT
	Reverse: ATAGTGGGGCAGGTCCTTCT
ACSL4 (Human)	Forward: GACATTTGCGTACTTTATTGTCGG
	Reverse: CGAAGTGTGTGACAGAGCGATA
LPCAT3 (Human)	Forward: CCAGGAGCTGAGCCTTAACA
	Reverse: AAAGCAAAGGGGTAACCCAGG
β-actin (Human)	Forward: CTTCGCGGGCGACGAT
	Reverse: CCACATAGGAATCCTTCTGACC
GPX4 (Rat)	Forward: CATTCCCGAGCCTTTCAACC
	Reverse: CACACGCAACCCCTGTACTT
ACSL4 (Rat)	Forward: CACCTTCGATCCCAGGAGATT
	Reverse: TCCGGGTTTGTCTGAAGTGG
LPCAT3 (Rat)	Forward: GCCTTAACAAGTTGGCGACG
	Reverse: AACTGGTGGCCGAAGTTGAA
β-actin (Rat)	Forward: CTGTGTGGATTGGTGGCTCT
	Reverse: AGCTCAGTAACAGTCCGCCT

### 2.10 Western blot

Protein samples were extracted from tissues or cells using RIPA lysis buffer (KeyGEN BioTECH, #KGB5203) supplemented with 1 mM PMSF (Amresco, #97064–898). Protein concentration was determined using a BCA protein assay kit (KeyGEN BioTECH, #KGB2101). Equal amounts of protein (30 μg per lane) were separated by 10% SDS-PAGE and transferred onto PVDF membranes (Millipore, #ISEQ00010). The membranes were blocked with 5% non-fat milk for 2 h at room temperature and then incubated overnight at 4 °C with the following primary antibodies: Anti-ACSL4 (Abcam, ab155282), Anti-LPCAT3 (AssayGenie, CAB17604), Anti-GPX4 (Abcam, ab125066), and Anti-β-actin (Abcam, ab8227). After washing, the membranes were incubated with HRP-conjugated Goat Anti-Rabbit IgG secondary antibody (Abcam, ab6721) for 2 h at room temperature. Protein bands were visualized using an ECL chemiluminescence kit (Thermo Fisher Scientific, #32209) and imaged with a Tanon 5,200 imaging system (Tanon Science & Technology Co., Ltd., China). Densitometric analysis was performed using ImageJ software (National Institutes of Health, USA).

### 2.11 Cell viability assay

Cell viability was assessed using a Cell Counting Kit-8 (CCK-8; Beyotime, #C0037, Shanghai, China). HTR-8/SVneo cells were seeded into 96-well plates at a density of 5 × 10^3^ cells per well and allowed to adhere overnight. Following the designated experimental treatments, 10 μL of CCK-8 reagent was added to each well. The plates were then incubated at 37 °C for 2 h in the dark. The absorbance of each well was measured at a wavelength of 450 nm using a full-wavelength microplate analyzer (HBS-SCANX, Nanjing Detie Biotechnology Co., Ltd., China). The results were normalized to the absorbance values of the control group to calculate the percentage of cell viability.

### 2.12 Mitochondrial membrane potential assay

The mitochondrial membrane potential (ΔΨm) of HTR-8/SVneo cells was evaluated using a JC-1 assay kit (Abcam, #ab113850, Cambridge, UK). Cells in the logarithmic growth phase were seeded into 96-well plates at a density of 1.5 × 10^4^ cells per well and incubated according to the experimental groupings. After treatment, cells were washed once with 100 μL of 1 × PBS per well. Subsequently, 100 μL of JC-1 working solution was added to each well, and the plates were incubated at 37 °C for 10 min in the dark. Following incubation, the cells were washed twice with the provided 1 × assay buffer. Fluorescence was measured using a full-wavelength microplate analyzer (HBS-SCANX, Nanjing Detie Biotechnology Co., Ltd., China) with excitation/emission wavelengths set at 530 nm/590 nm for J-aggregates and 485 nm/530 nm for JC-1 monomers. The ratio of aggregate to monomer fluorescence intensity was calculated to assess changes in mitochondrial membrane potential.

### 2.13 Reactive oxygen species (ROS)

Intracellular ROS levels in HTR-8/SVneo cells were measured using a Reactive Oxygen Species Assay Kit (Beyotime, #S0033S, Shanghai, China). The fluorescent probe DCFH-DA was diluted 1:1000 in serum-free culture medium to a final concentration of 10 μM. After experimental treatments, cells were collected and resuspended in the diluted DCFH-DA solution at a density of 1 × 10^6^ cells/mL, followed by incubation at 37 °C for 20 min in the dark. To remove any unincorporated DCFH-DA, the cells were washed three times with serum-free medium. Fluorescence intensity was then measured using a microplate analyzer (HBS-SCANX, Nanjing Detie Biotechnology Co., Ltd., China) with excitation and emission wavelengths set at 485 nm and 520 nm, respectively.

ROS levels in placental tissues were assessed using dihydroethidium (DHE, Thermo Fisher Scientific, #D1168, Pittsburgh, PA, USA) fluorescence staining. Fresh-frozen or paraffin-embedded placental sections were brought to room temperature for 30 min. After washing with PBS, sections were incubated with 2 μmol/L DHE working solution (prepared in DMSO) at 37 °C for 30 min in the dark. Sections were then rinsed with PBS to remove unbound dye and counterstained with DAPI (Thermo Fisher Scientific, #D1306) for nuclear visualization. After mounting with antifade medium, images were captured under a fluorescence microscope (DM4B, Leica Microsystems, Germany) at 400 × magnification.

### 2.14 Quantification of short-chain fatty acids (SCFAs) by gas chromatography–mass spectrometry (GC–MS)

The concentrations of SCFAs, including acetic acid, propionic acid, and butyric acid, in colonic content homogenates were determined by GC–MS. Sample preparation was performed as previously described with modifications. Briefly, intestinal content samples were homogenized in normal saline at a ratio of 1:5 (w/v). 50 μL of the resulting homogenate was transferred to a microcentrifuge tube for derivatization.

A Shimadzu GC/MS QP2010 Ultra system (Shimadzu Corporation, Kyoto, Japan) equipped with an Rtx-5MS capillary column (30 m × 0.25 mm ID, 0.25 μm film thickness, Restek Corporation, USA) was used for analysis. The GC conditions were as follows: injection volume 1 μL, split ratio 1:10, helium carrier gas at a constant flow rate of 1.5 mL/min. The temperature program began at 80 °C (held for 3 min), ramped to 300 °C at 20 °C/min, and held for 5 min. The injector liner temperature was set to 250 °C, and the purge flow rate was 20 mL/min for 1 min.

Mass spectrometric detection was conducted in electron impact (EI) mode at 70 eV. The ion source and transfer line temperatures were set to 200 °C and 220 °C, respectively. The detector voltage was −950 V. Quantification was performed using selected ion monitoring (SIM) mode with the following ion transitions: acetic acid (m/z 60.0 to 43.1), propionic acid (m/z 74.0 to 55.0), and butyric acid (m/z 60.0 to 42.1). The solvent delay time was set to 300 s.

Quantification was achieved using external standard curves. Individual SCFA standard stock solutions (5 mol/L) were serially diluted to generate calibration standards ranging from 0.1 to 100 μmol/L. These standards were processed identically to the samples, including a derivatization step with ethyl acetate. The lower limit of detection (LLOD) for all analytes was 0.1 μmol/L. The peak areas for the quantitative ions were used to construct the standard curves, and the concentrations in unknown samples were calculated by interpolation from these curves.

### 2.15 Quantification of polyunsaturated fatty acids (PUFAs) by gas chromatography–mass spectrometry (GC–MS)

The concentrations of key polyunsaturated fatty acids (PUFAs), including arachidonic acid (AA), adrenic acid (AdA), docosahexaenoic acid (DHA), and eicosapentaenoic acid (EPA), in placental tissue and cell homogenates were determined using gas chromatography–mass spectrometry (GC–MS). Sample preparation followed a derivatization protocol to enhance volatility and detection sensitivity.

Briefly, placental tissue or cell samples were homogenized in normal saline at a ratio of 1:5 (w/v). A 50 μL aliquot of the homogenate was transferred to a microcentrifuge tube for derivatization. The detailed derivatization procedure involved methoxylation and silylation steps: after adding 150 μL methanol and vortexing, the mixture was centrifuged at 18,000 rpm for 5 min. The supernatant was collected, vacuum-dried, and then derivatized with 30 μL methoxyamine hydrochloride in pyridine (10 mg/mL) for 24 h at room temperature, followed by the addition of 30 μL MSTFA (containing 1% TMCS) and 30 μL n-heptane. The final mixture was vortexed for 1 min and 1 μL was injected into the GC–MS system.

Analysis was performed using a Shimadzu GC/MS QP2010 Ultra system (Shimadzu Corporation, Kyoto, Japan) equipped with an Rtx-5MS capillary column (30 m × 0.25 mm ID, 0.25 μm film thickness, Restek Corporation, USA). The GC conditions were as follows: injection volume 1 μL, split ratio 1:10, helium carrier gas at a constant flow rate of 1.5 mL/min. The temperature program began at 80 °C (held for 3 min), ramped to 300 °C at 20 °C/min, and held for 5 min. The injector liner temperature was set to 250 °C, and the purge flow rate was 20 mL/min for 1 min.

Mass spectrometric detection was conducted in electron impact (EI) mode at 70 eV. The ion source and transfer line temperatures were set to 200 °C and 220 °C, respectively. The detector voltage was −950 V. Quantification was performed using selected ion monitoring (SIM) mode with the following ion transitions: m/z 79.0 for AA and EPA, m/z 226.0 for AdA, and m/z 198.0 for DHA. The solvent delay time was set to 300 s.

Calibration curves were constructed using external standards. Individual PUFA standard stock solutions (5 mM) were serially diluted to concentrations ranging from 0.1 to 1,000 μM. These standards underwent the same derivatization process as the samples. The lower limit of detection (LLOD) for all PUFAs was 0.1 μM. Quantification was achieved by interpolating peak areas of the target ions from the standard curves.

All sample concentrations were normalized to protein content or cell number as appropriate.

### 2.16 Microbial 16S rRNA gene sequencing and bioinformatic analysis

Fecal samples were collected from the colonic contents of rats in each experimental group. Microbial genomic DNA was extracted, and the V3–V4 hypervariable region of the 16S rRNA gene was amplified using specific primers (338F, 5′-ACTCCTACGGGAGGCAGCAG-3′ and 806R, 5′-GGACTACHVGGGTWTCTAAT-3′). PCR reactions were carried out in a 15 μL mixture containing Phusion High-Fidelity PCR Master Mix, 0.2 μM of each primer, and approximately 10 ng of template DNA. Thermal cycling conditions were as follows: initial denaturation at 98 °C for 1 min; 30 cycles of denaturation at 98 °C for 10 s, annealing at 50 °C for 30 s, and elongation at 72 °C for 30 s; followed by a final extension at 72 °C for 5 min (Magoc and Salzberg, 2011). PCR products were purified using magnetic bead purification, quantified, and pooled in equimolar ratios. Sequencing libraries were constructed, and paired-end sequencing (2 × 250 bp) was performed on an Illumina NovaSeq 6,000 platform (Illumina, USA) according to standard protocols.

Raw sequencing data were processed using the QIIME2 pipeline (version 2023.2). Paired-end reads were demultiplexed and quality-filtered using fastp (v0.23.1) to obtain high-quality clean tags (Bokulich et al., 2012). Sequences were merged using FLASH (v1.2.11) to generate raw tags (Magoc and Salzberg, 2011). Chimeric sequences were identified and removed using the vsearch plugin (v2.16.0) against the SILVA database (v138) (Edgar et al., 2011). Denoising was performed using DADA2 to obtain amplicon sequence variants (ASVs). Taxonomic assignment of ASVs was performed using a pre-trained Naïve Bayes classifier based on the SILVA 138 reference database. For sequences that could not be annotated using the Silva taxonomic file, the NCBI taxonomic database was used to supplement taxonomic information via the LCA (Least Common Ancestor) algorithm.

Alpha diversity indices (Chao1, Shannon, Simpson, Observed ASVs, Dominance, Good’s coverage, Pielou_e) were calculated to assess microbial richness, diversity, and evenness within samples. Beta diversity was evaluated using weighted and unweighted UniFrac distances and visualized via principal coordinate analysis (PCoA), non-metric multidimensional scaling (NMDS), and cluster analysis. Differential abundance analysis between groups was performed using ANCOM and LEfSe to identify significantly altered taxa. Functional potential of the microbial communities was predicted using PICRUSt2 (v2.3.0) (Douglas et al., 2020). All statistical analyses and visualizations were performed using R (v4.0.3) with packages including vegan, ggplot2, metagenomeSeq, and lefse.

### 2.17 Data analysis and statistics

Normality was assessed using the Shapiro–Wilk test, and homogeneity of variances was assessed using Levene’s test. For two-group comparisons, Student’s *t*-test was used for normally distributed data, whereas the Mann–Whitney U test was used for non-normally distributed data. For comparisons involving more than two groups, overall differences were first assessed using one-way analysis of variance (ANOVA), followed by *post hoc* tests selected according to the comparison of interest. Dunnett’s *post hoc* test was used for comparisons of each treatment group (Model or Abx) with the common control group (NC), whereas Tukey’s honest significant difference test was used for direct pairwise comparison between the Model and Abx groups. When the assumptions for ANOVA were not met, the Kruskal-Wallis test followed by Dunn’s multiple-comparisons test was applied. Correlation analyses between microbial taxa and SCFA levels were performed using Pearson correlation for normally distributed data or Spearman’s rank correlation for non-normally distributed data. Data are presented as mean ± SD from at least three independent replicates. All statistical tests were two-sided, and significance was defined as ^*^p < 0.05, ^**^p < 0.01, and ^***^p < 0.001. GraphPad Prism and ImageJ were used for data analysis and visualization, and figures were assembled in Adobe Illustrator.

## 3 Results

### 3.1 GDM and antibiotic-induced dysbiosis lead to metabolic dysfunction and placental injury

**Figure 2:**
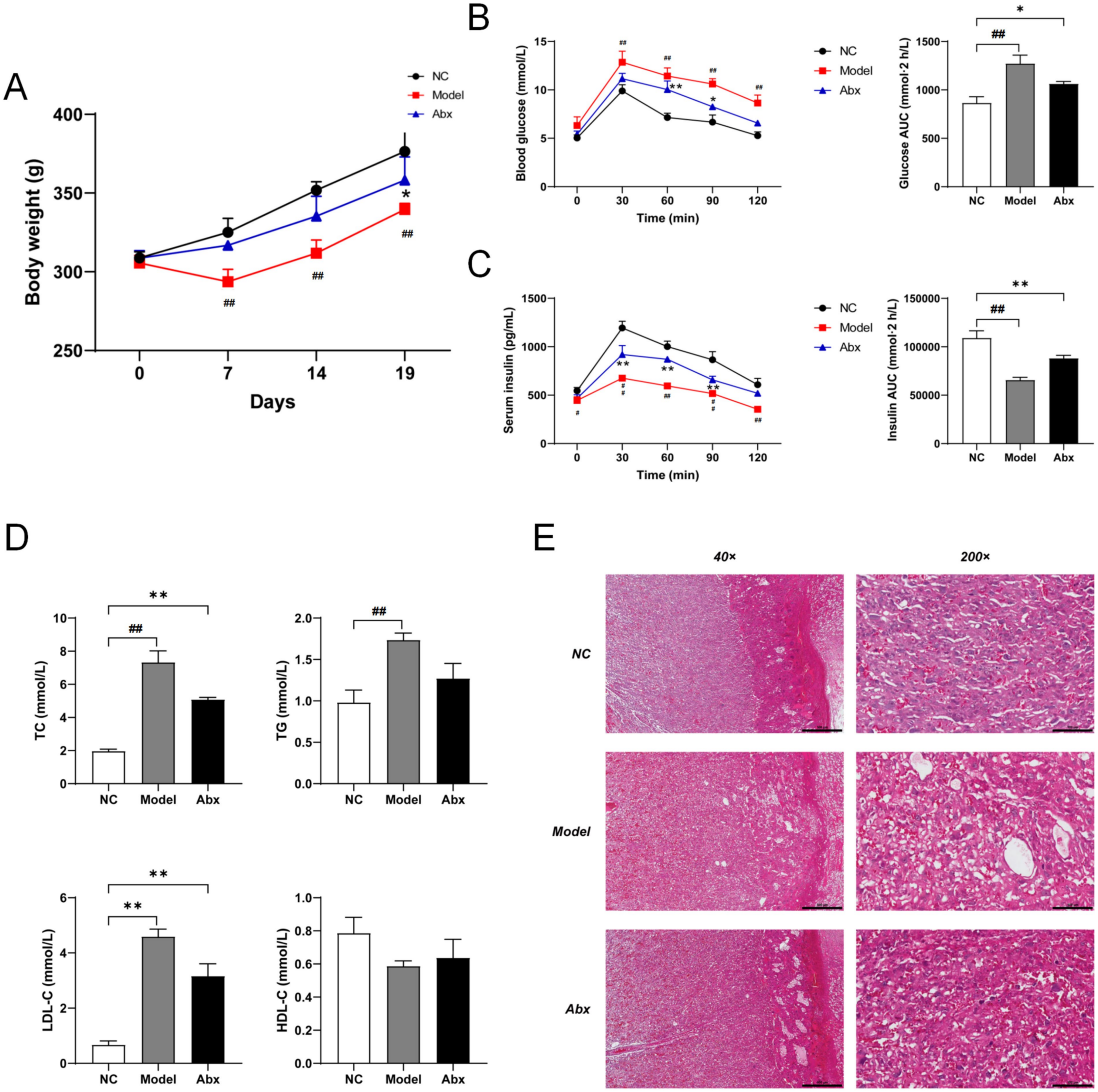
Evaluation of metabolic phenotypes and placental histoarchitecture in experimental rats. **(A)** Body weight changes of rats in the NC, model, and ABX groups throughout gestation (days 0, 7, 14, and 19). **(B)** Blood glucose levels during the oral glucose tolerance test (OGTT) performed on gestational day 19 and area under the curve (AUC) for the OGTT. **(C)** Serum insulin levels during the insulin release test and AUC for the insulin release test. **(D)** Serum lipid profiles, including total cholesterol (TC), triglycerides (TG), low-density lipoprotein cholesterol (LDL-C), and high-density lipoprotein cholesterol (HDL-C). **(E)** Representative hematoxylin and eosin (H&E)-stained images of placental tissues (scale bars: 500 μm for 40 × magnification; 100 μm for 200 × magnification). Statistical comparisons for time-course data (A, B-left, C-left) were performed using two-way ANOVA, while single-point comparisons (B-right, C-right, D) were analyzed using one-way ANOVA, both followed by Dunnett’s *post hoc* test. *p < 0.05, **p < 0.01 NC vs. A group; ^#^*p* < 0.05, ^##^*p* < 0.01 NC vs. odel group.

To investigate the systemic metabolic alterations induced by GDM and gut microbiota depletion, we first monitored the physiological and metabolic parameters in rats. Compared to the NC group, rats in the Model group exhibited a significant reduction in body weight gain during pregnancy (*p* < 0.05; Figure 2A). Similarly, administration of the Abx resulted in a notable decrease in body weight, with the difference becoming statistically significant in the late gestational period (*p* < 0.05; Figure 2A).

We next assessed glucose homeostasis. Following an oral glucose challenge, both the Model and Abx groups displayed significantly elevated blood glucose levels at all time points compared to the NC group. Consequently, the total AUC for glucose was markedly increased in these two groups (*p* < 0.01; Figure 2B). Concordantly, the insulin secretory capacity was severely impaired in the Model and Abx groups, as evidenced by significantly lower serum insulin levels during the test (*p* < 0.05) and a corresponding reduction in the insulin AUC (*p* < 0.01; Figure 2C).

Dyslipidemia, a hallmark of metabolic syndrome, was also evident. Serum levels of TC were significantly elevated in the Model group (*p* < 0.05), while TG and LDL-C levels were even more markedly increased (*p* < 0.01; Figure 2D; Supplementary Table S1). While the Abx group also showed a trend towards increased TC and LDL-C, the changes in TG and HDL-C did not reach statistical significance (Figure 2D).

Finally, histological examination of placental tissue revealed substantial morphological disruption. Placental tissues from the NC group showed clear stratification, orderly cell arrangement, and uniform interstitial spaces. In contrast, the Model group exhibited obscured tissue boundaries, disorganized and loosely distributed cells, and enlarged intercellular spaces. The Abx group also displayed milder but noticeable histological abnormalities, including blurred laminar boundaries and disordered cell arrangement compared to the NC group (Figure 2E).

Collectively, these results demonstrate that both STZ-induced GDM and antibiotic-induced microbiota depletion successfully recapitulate key features of metabolic dysfunction, including impaired glucose tolerance, insulin resistance, dyslipidemia, and concomitant placental injury.

### 3.2 GDM and antibiotic treatment induce gut dysbiosis characterized by depletion of SCFA-producing taxa and reduced SCFA levels

**Figure 3:**
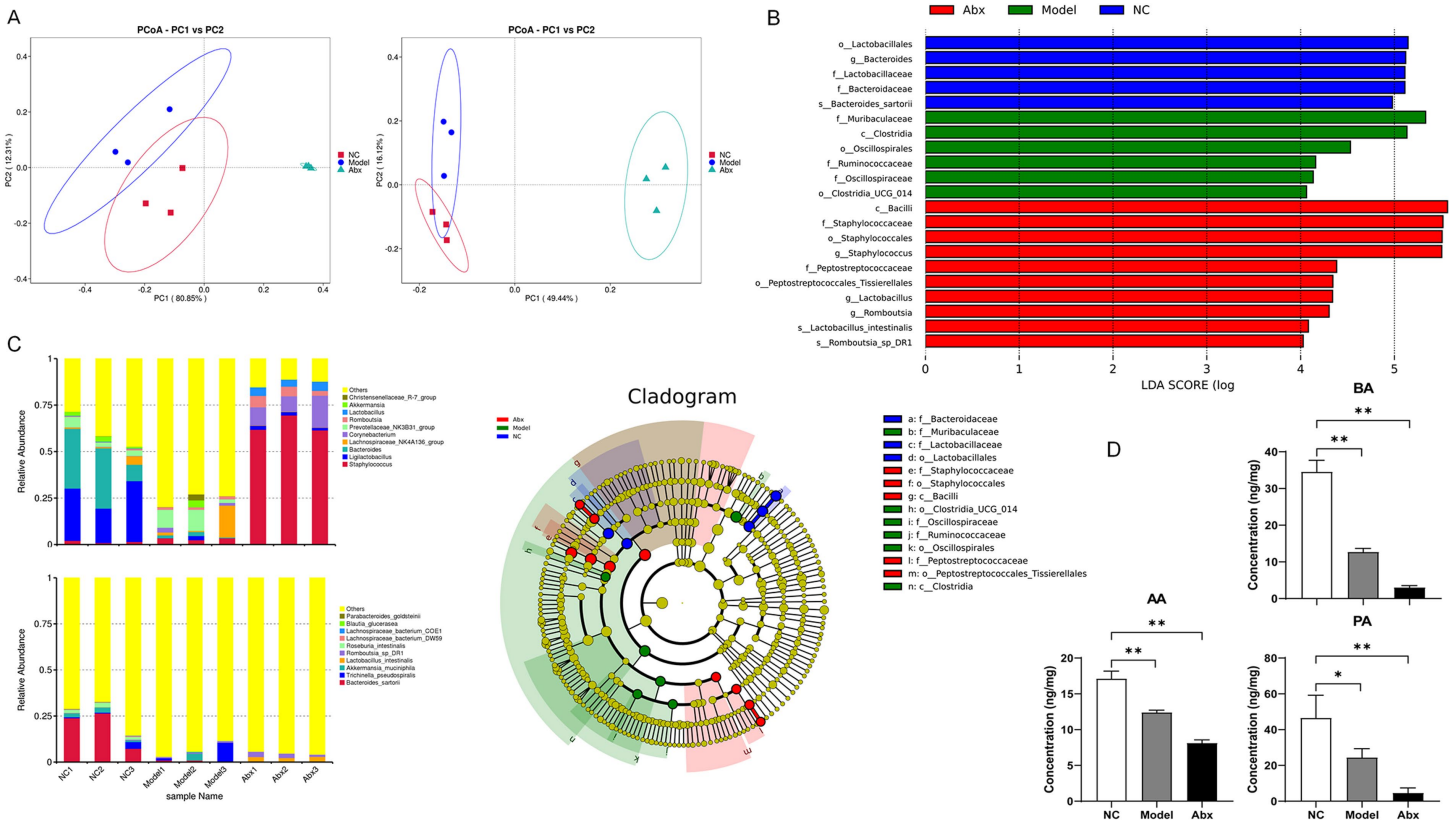
Gut microbiota dysbiosis and reduction of microbial metabolites SCFAs in GDM and antibiotic-treated rats. **(A)** Principal coordinate analysis (PCoA) based on *β*-diversity showing distinct clustering of gut microbial communities in the NC (normal control), model, and Abx (antibiotic-treated) groups. Percentages on the axes indicate the proportion of variance explained by each coordinate. Ellipses represent the 95% confidence intervals of each group. **(B)** Linear discriminant analysis effect size (LEfSe) identifying discriminative taxa among the three groups. The histogram shows the linear discriminant analysis (LDA) scores (log10) of significantly enriched taxa, and the cladogram highlights their phylogenetic positions. Only taxa meeting the threshold (LDA score > 2.0, *p* < 0.05) are shown. **(C)** Relative abundance of gut microbial taxa at the genus and/or species level. Stacked bar plots display the average relative abundances of the top taxa across groups, with low-abundance taxa pooled as others. **(D)** Concentrations of short-chain fatty acids (SCFAs), including acetate, propionate, and butyrate, in colonic contents as determined by GC–MS. Data are presented as mean ± SD (*n* = 3). For GC–MS measurements of SCFAs, three rats were randomly selected from each experimental group (*n* = 12 per group for the main study) **(D)** were compared using a one-way ANOVA followed by Dunnett’s *post hoc* test. *p < 0.05, **p < 0.01 NC vs. Abx or odel group.

The gut microbiota structure was profoundly altered in both pathological and intervention models. PCoA based on Weighted UniFrac distance revealed a clear separation among the NC, Model, and Abx groups, indicating that both GDM and antibiotic intervention significantly reshaped the overall gut ecosystem (Figure 3A). The microbial profile of the Abx group was the most distinct, clustering far from the other groups. The Model group exhibited an intermediate state between the NC and Abx groups, suggesting GDM induces a significant dysbiosis.

LEfSe analysis identified specific bacterial taxa driving these community differences (Figure 3B). The NC group was characterized by a significant enrichment of *Lactobacillales* and *Lactobacillaceae*. In contrast, the Model group showed a marked increase in *Muribaculaceae* and *Oscillospiraceae*. The Abx group displayed a unique signature with enrichment of *Bacteroides* and *Lactobacillaceae*.

Crucially, we observed a concerted depletion of key SCFA-producing taxa in the Model and Abx groups (Figure 3C). At the genus level, the abundance of *Lachnospiraceae_NK4A136_group*, a known butyrate producer, was significantly reduced. This was corroborated at the species level by a severe reduction in *Roseburia intestinalis* and *Blautia glucerasea*, both renowned for their ability to produce butyrate and acetate. Concurrently, although not direct SCFA producers, beneficial mucin-degrading bacteria like *Akkermansia muciniphila* were also diminished. The relative abundances and statistical significance of these key taxa are detailed in Table 2.

**Table 2:** Relative abundance of key SCFA-producing and beneficial gut microbial taxa in experimental groups.

Taxon	NC group (% relative abundance)	Model group (% relative abundance)	Abx group (% relative abundance)	*p*-value (Model vs. NC)	*p*-value (Abx vs. NC)
*Lachnospiraceae_NK4A136_group*	5.25 ± 0.82	1.53 ± 0.31	0.78 ± 0.18	< 0.001	< 0.001
*Roseburia intestinalis*	3.12 ± 0.48	0.87 ± 0.22	0.45 ± 0.12	< 0.001	< 0.001
*Blautia glucerasea*	2.84 ± 0.42	1.02 ± 0.26	0.61 ± 0.15	< 0.01	< 0.001
*Akkermansia muciniphila*	4.56 ± 0.71	1.18 ± 0.35	0.72 ± 0.24	< 0.001	< 0.001

Data are presented as mean ± SD (*n* = 12 per group). Relative abundance was calculated from 16S rRNA gene sequencing data. *p*-values were derived from one-way ANOVA followed by Dunnett’s *post hoc* test comparing each treatment group to the NC group. All taxa showed significant reduction in the Model and Abx groups compared to the NC group, consistent with the described depletion of SCFA-producing bacteria.

This targeted loss of SCFA-producing functionality was directly confirmed by biochemical measurement. GC–MS analysis of colonic contents revealed that the concentrations of acetate, propionate, and butyrate were significantly decreased in both the Model and Abx groups compared to the NC controls (Figure 3D; Supplementary Table S1; *p* < 0.05). Furthermore, to quantitatively link the microbial dysbiosis to the metabolic outcome, we performed a correlation analysis which demonstrated strong positive associations between the abundance of key SCFA-producing bacteria and the levels of colonic SCFAs (Table 3).

**Table 3:** Correlation analysis between key microbial taxa and short-chain fatty acid (SCFA) levels.

Microbial Taxon	Acetate (r)	Acetate (p)	Propionate (r)	Propionate (p)	Butyrate (r)	Butyrate (p)
*Lachnospiraceae_NK4A136_group*	0.82	< 0.001	0.75	< 0.001	0.89	< 0.001
*Roseburia intestinalis*	0.79	< 0.001	0.71	< 0.001	0.85	< 0.001
*Blautia glucerasea*	0.76	< 0.001	0.68	< 0.001	0.81	< 0.001
*Akkermansia muciniphila*	0.65	< 0.01	0.58	< 0.01	0.72	< 0.001

Pearson correlation analysis (r: correlation coefficient) was performed using data from all animals across the NC, Model, and Abx groups (*n* = 36). All four taxa showed significant positive correlations with the concentrations of all three major SCFAs, reinforcing the functional role of these bacteria in SCFA production.

In summary, these results demonstrate that GDM and antibiotic treatment induce distinct microbial dysbiosis, which converges on a common functional outcome: the depletion of pivotal SCFA-producing bacteria and a consequent deficiency in colonic SCFAs.

### 3.3 Depletion of gut microbiota and SCFAs exacerbates placental ferroptosis and inflammation

**Figure 4:**
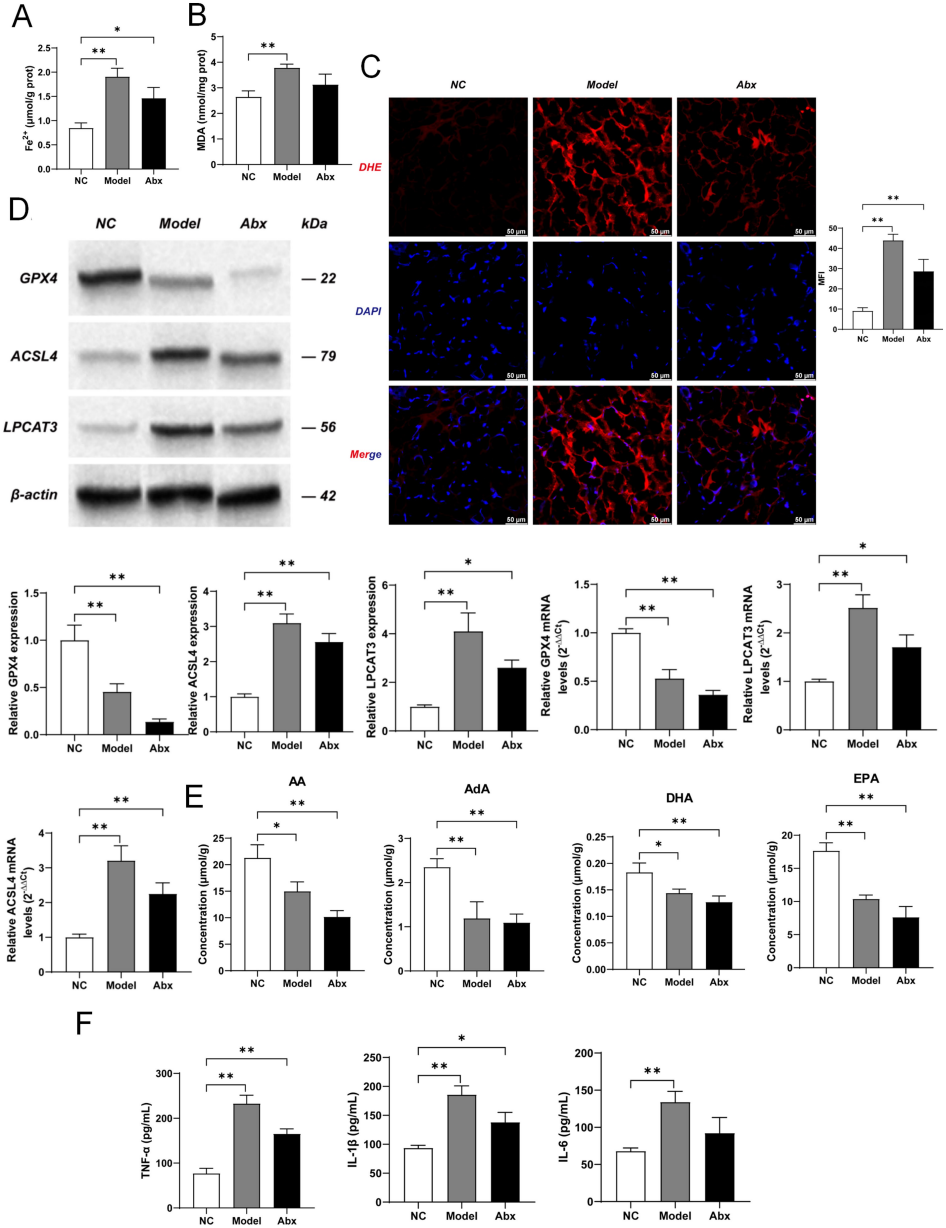
GDM and antibiotic-induced dysbiosis trigger placental ferroptosis and inflammatory responses. **(A)** Placental Fe^2+^ content. **(B)** Placental malondialdehyde (MDA) content, a marker of lipid peroxidation. **(C)** Representative images (scale bars: 50 μm) and quantitative analysis of reactive oxygen species (ROS) in placental tissues detected by DHE staining (red fluorescence). **(D)** Representative western blot bands and quantitative analysis of GPX4, ACSL4, and LPCAT3 protein and mRNA expression and in placental tissues. β-Actin was used as a loading control. **(E)** Contents of polyunsaturated fatty acids (PUFAs), including arachidonic acid (AA), adrenic acid (AdA), docosahexaenoic acid (DHA), and eicosapentaenoic acid (EPA), in placental tissues as measured by GC–MS. **(F)** Serum levels of inflammatory cytokines TNF-*α*, IL-6, and IL-1β as determined by ELISA. Data are presented as mean ± SD (*n* = 3). All quantitative comparisons were performed using one-way ANOVA followed by Dunnett’s *post hoc* test. *p < 0.05, **p < 0.01 NC vs. ABX or Model group.

Having established that GDM and antibiotic treatment lead to a deficiency in SCFAs, we next investigated the downstream consequences on placental homeostasis. We found that both the Model and Abx groups exhibited a significant accumulation of Fe^2+^ in placental tissue compared to the NC group (*p* < 0.01; Figure 4A; Supplementary Table S1), indicating iron dysregulation. Concordantly, the level of MDA, a terminal product of lipid peroxidation, was markedly elevated in both the Model group (p < 0.01) and the Abx group (*p* < 0.05) (Figure 4B; Supplementary Table S1). This was further supported by a substantial increase in ROS, as visualized by intense DHE fluorescence in the placental sections of the Model and Abx groups (*p* < 0.01; Figure 4C; Supplementary Table S1). These results collectively demonstrate that placental tissue undergoes severe oxidative stress and lipid peroxidation, hallmarks of ferroptosis.

To elucidate the molecular mechanism underlying this process, we examined the expression of key regulators of ferroptosis. Western blot and qPCR analyses revealed a consistent downregulation of the antioxidant enzyme GPX4 at both the protein and mRNA levels in the Model and Abx groups (*p* < 0.01; Figure 4D; Supplementary Table S1). Conversely, the expression of ACSL4 and LPCAT3, two critical enzymes that prime phospholipids for peroxidation, was significantly upregulated (p < 0.01 for ACSL4 protein and mRNA; *p* < 0.01 for LPCAT3 protein and mRNA; Figure 4D; Supplementary Table S1). This coordinated molecular shift, suppression of GPX4 and induction of ACSL4/LPCAT3, creates a cellular environment highly susceptible to ferroptosis.

We then assessed the content of phospholipid substrates. GC–MS analysis showed that the placental levels of key PUFAs, including AA, AdA, DHA, and EPA, were substantially depleted in the diseased and treated groups (*p* < 0.01 for AA and AdA; *p* < 0.05 for DHA and EPA in both Model and Abx groups vs. NC; Figure 4E; Supplementary Table S1). This reduction in PUFA substrates is consistent with their excessive consumption during ACSL4/LPCAT3-mediated lipid peroxidation.

Finally, we assessed the systemic inflammatory response. ELISA results confirmed that the serum concentrations of pro-inflammatory cytokines TNF-α (*p* < 0.01), IL-6 (*p* < 0.01), and IL-1β (*p* < 0.01) were significantly elevated in the Model group. The Abx group also showed a significant increase in TNF-α (*p* < 0.01) and IL-1β (*p* < 0.01), with a non-significant trend for IL-6 (Figure 4F; Supplementary Table S1), indicating that both GDM and microbial disruption induce a strong inflammatory state.

In summary, these data illustrate a coherent pathway: the loss of gut microbiota and SCFAs promotes a ferroptotic cascade in the placenta, characterized by iron overload, GPX4 suppression, ACSL4/LPCAT3 activation, PUFA depletion, and massive lipid peroxidation, ultimately culminating in systemic inflammation.

### 3.4 SCFAs protect against insulin resistance-induced trophoblast dysfunction by suppressing the ACSL4/LPCAT3-mediated ferroptotic pathway *in vitro*

**Figure 5:**
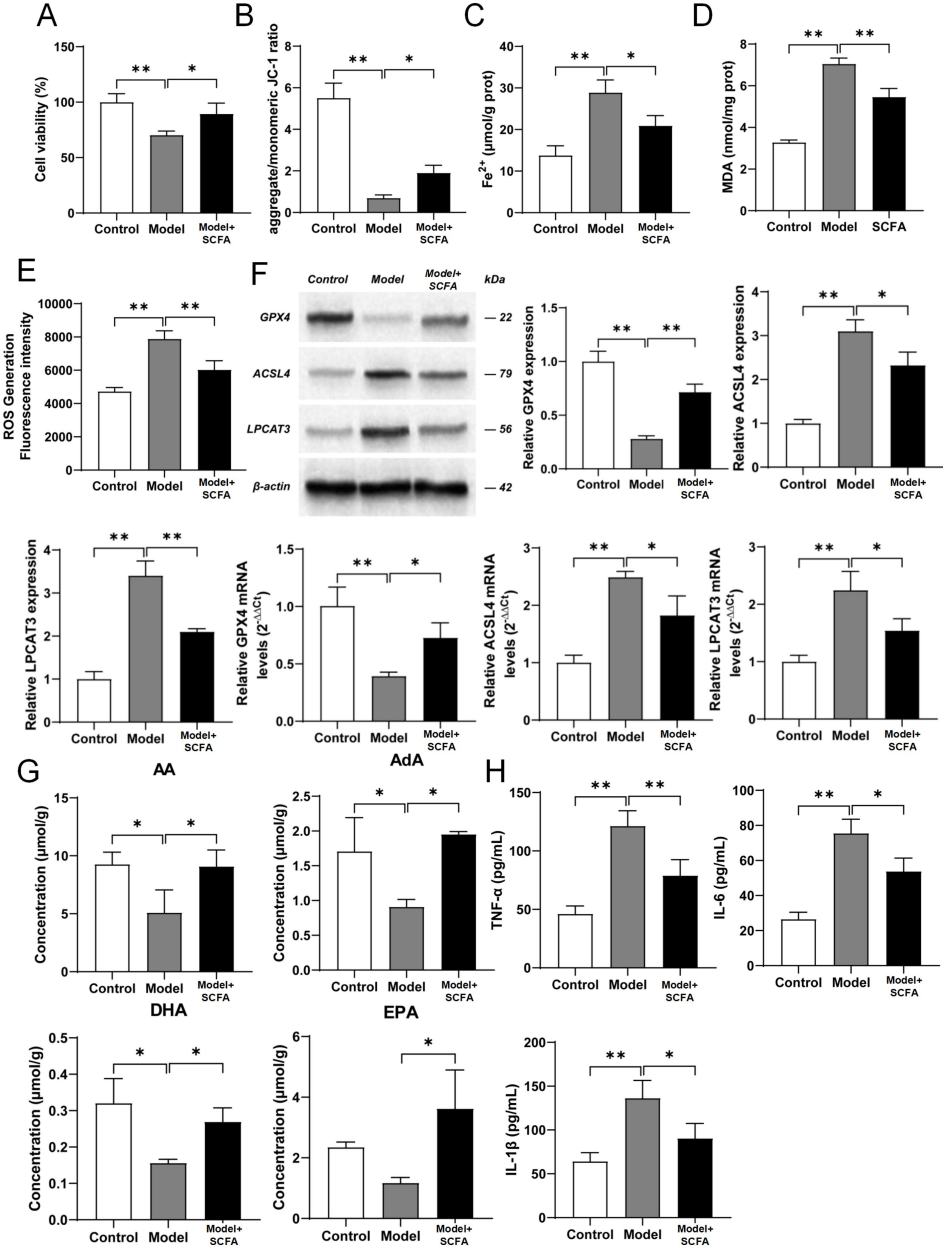
SCFA treatment ameliorates insulin resistance-induced ferroptosis and inflammation in HTR-8/SVneo trophoblast cells. **(A)** Cell viability assessed by CCK-8 assay. **(B)** Mitochondrial membrane potential measured by JC-1 assay (ratio of aggregate/monomer fluorescence). **(C)** Intracellular Fe^2+^ content. **(D)** Lipid peroxidation level indicated by MDA content. **(E)** Intracellular ROS levels detected by DCFH-DA probe. **(F)** Representative western blot bands and quantitative analysis of GPX4, ACSL4, and LPCAT3 mRNA and protein expression. β-Actin was used as a loading control. **(G)** Contents of polyunsaturated fatty acids (PUFAs), including AA, AdA, DHA, and EPA, in cells as measured by GC–MS. **(H)** Levels of inflammatory cytokines TNF-α, IL-6, and IL-1β in the cell culture supernatant as determined by ELISA. Data are presented as mean ± SD (*n* = 6). All quantitative comparisons were performed using one-way ANOVA followed by Dunnett’s *post hoc* test. *p < 0.05, **p < 0.01 Model vs. Control or Model + SCFA group.

To establish a direct causal link between SCFAs and placental ferroptosis, we utilized an in vitro model of insulin resistance in human trophoblast cells (HTR-8/SVneo). Treatment with high-dose insulin (1 × 10^−6^ M) for 48 h successfully induced cellular dysfunction, as evidenced by a significant decrease in cell viability (*p* < 0.01; Figure 5A; Supplementary Table S1) and a collapse of mitochondrial membrane potential (*p* < 0.01; Figure 5B; Supplementary Table S1). This was accompanied by hallmark features of ferroptosis: a dramatic accumulation of intracellular Fe^2+^ (*p* < 0.01; Figure 5C; Supplementary Table S1), elevated MDA levels (*p* < 0.01; Figure 5D; Supplementary Table S1), and a surge in ROS production (*p* < 0.01; Figure 5E; Supplementary Table S1).

Critically, co-treatment with a physiological mixture of SCFAs (5 mM) largely rescued these deleterious effects. SCFA supplementation significantly restored cell viability (*p* < 0.05 vs. Model group; Figure 5A; Supplementary Table S1), stabilized mitochondrial membrane potential (*p* < 0.01 vs. Model group; Figure 5B; Supplementary Table S1), and markedly attenuated iron overload (*p* < 0.05), lipid peroxidation (*p* < 0.01), and oxidative stress (*p* < 0.01) when compared to the Model group (Figures 5C–E; Supplementary Table S1).

At the molecular level, the insulin-resistant model recapitulated the key findings from our animal study. The expression of the protective enzyme GPX4 was suppressed at both protein and mRNA levels (*p* < 0.01; Figure 5F; Supplementary Table S1), while the pro-ferroptotic enzymes ACSL4 and LPCAT3 were significantly upregulated (*p* < 0.01; Figure 5F; Supplementary Table S1). Once again, SCFA treatment effectively reversed this expression pattern, promoting GPX4 expression and suppressing ACSL4 and LPCAT3 expression at both the protein (*p* < 0.01) and mRNA (*p* < 0.05) levels vs. Model group (Figure 5F; Supplementary Table S1).

We also observed that PUFA substrates (AA, AdA, DHA, EPA) were consumed in the Model group, consistent with heightened lipid peroxidation (*p* < 0.05; Figure 5G; Supplementary Table S1). SCFA treatment restored the cellular levels of these PUFAs (*p* < 0.05 vs. Model group; Figure 5G; Supplementary Table S1). Finally, insulin resistance triggered a robust inflammatory response, with secretion of TNF-α, IL-6, and IL-1β into the culture medium being significantly increased (*p* < 0.01; Figure 5H; Supplementary Table S1). This pro-inflammatory state was also mitigated by SCFA co-treatment (*p* < 0.01 for TNF-α, *p* < 0.05 for IL-6 and IL-1β vs. Model group; Figure 5H; Supplementary Table S1).

In conclusion, these *in vitro* data mechanistically demonstrate that SCFAs directly attenuate insulin resistance-induced trophoblast injury by inhibiting the ACSL4/LPCAT3 pathway, thereby preventing ferroptosis and its associated inflammatory response.

## 4 Discussion

### 4.1 Principal findings and the gut-placenta axis

**Figure 6:**
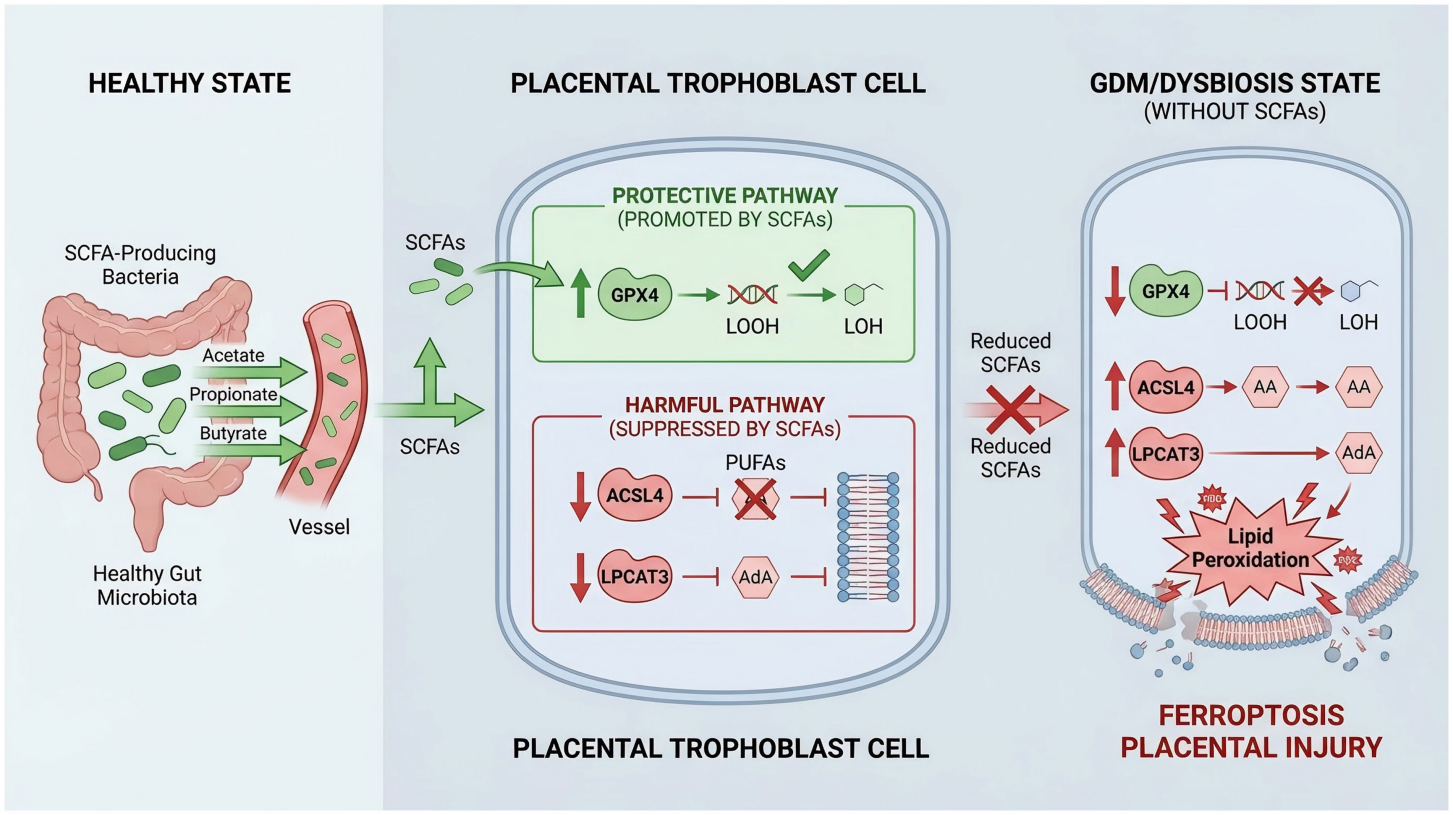
Proposed mechanism by which gut microbiota-derived short-chain fatty acids (SCFAs) protect against gestational diabetes mellitus (GDM)-induced placental ferroptosis. (Left panel) In the healthy state, SCFA-producing bacteria in the gut microbiota generate acetate, propionate, and butyrate, which enter the bloodstream and reach placental trophoblast cells. (Center panel) Within placental trophoblast cells, SCFAs regulate two opposing pathways: (1) Protective pathway (green box): SCFAs upregulate glutathione peroxidase 4 (GPX4) expression, which reduces lipid hydroperoxides (LOOH) to lipid alcohols (LOH), thereby preventing lipid peroxidation and maintaining membrane integrity. (2) Harmful pathway (red box): SCFAs suppress the expression of acyl-CoA synthetase long-chain family member 4 (ACSL4) and lysophosphatidylcholine acyltransferase 3 (LPCAT3), thereby preventing the activation and incorporation of polyunsaturated fatty acids [PUFAs, including arachidonic acid (AA) and adrenic acid (AdA)] into membrane phospholipids, which would otherwise be vulnerable to peroxidation. (Right panel) In the GDM/dysbiosis state, reduced SCFA production disrupts this protective mechanism. Downregulation of GPX4 impairs the antioxidant defense, while upregulation of ACSL4 and LPCAT3 promotes PUFA incorporation into membrane phospholipids. The resulting accumulation of lipid peroxides triggers ferroptosis and subsequent placental injury. SCFAs, short-chain fatty acids; GDM, gestational diabetes mellitus; GPX4, glutathione peroxidase 4; ACSL4, acyl-CoA synthetase long-chain family member 4; LPCAT3, lysophosphatidylcholine acyltransferase 3; PUFAs, polyunsaturated fatty acids; AA, arachidonic acid; AdA, adrenic acid; LOOH, lipid hydroperoxide; LOH, lipid alcohol.

This study suggests that gut microbiota-derived SCFAs may exert protective effects against placental trophoblast injury and insulin resistance in GDM, potentially in association with suppression of ACSL4/LPCAT3-related ferroptotic signaling. Our integrated approach, combining *in vivo* and in vitro findings support a possible mechanistic link that GDM-associated gut dysbiosis is accompany by reduced SCFAs levels and ferroptosis cascade, including downregulation of GPX4 and upregulation of ACSL4/LPCAT3, resulting in lipid peroxidation, cellular damage, and metabolic dysfunction (Figure 6).

A key finding of our study is the suggestion of a novel gut-placenta axis wherein microbial metabolites directly govern placental cell fate. We first confirmed that both STZ-induced GDM and antibiotic-induced dysbiosis successfully recapitulated key clinical features of metabolic syndrome in pregnant rats, including glucose intolerance, insulin resistance, and dyslipidemia, consistent with previous clinical observations (Lin et al., 2025). More importantly, we found that this metabolic dysfunction was paralleled by a profound reshaping of the gut microbial ecosystem, characterized by a depletion of pivotal SCFA-producing bacteria such as *Lachnospiraceae_NK4A136_group* and *Roseburia intestinalis*. This specific loss of function translated into a significant reduction in the colonic levels of acetate, propionate, and butyrate. This finding aligns with a growing body of literature reporting gut microbiota alterations and SCFA deficiency in patients with GDM (Crusell et al., 2018; Qin et al., 2025). Our results extend these observations by establishing a direct correlation between the loss of these beneficial metabolites and the pathogenesis of placental injury.

### 4.2 Mechanistic insight: SCFA deficiency activates placental ferroptosis

Our findings are consistent with the hypothesis that SCFA deficiency unleashes ferroptosis in placental trophoblasts. We observed the classic hallmarks of ferroptosis in the placentas of our diseased models: iron overload, elevated lipid peroxidation (MDA), and massive ROS accumulation (Federico et al., 2025). At the molecular level, this was driven by a coordinated downregulation of the antioxidant enzyme GPX4 and a marked upregulation of the lipid-peroxidation enzymes ACSL4 and LPCAT3. This shift created a cellular environment primed for peroxidative damage, which was further substantiated by the significant consumption of key PUFA substrates (AA, AdA, DHA, EPA) (Zhang et al., 2024). This depletion of free PUFAs may reflect of their enzymatic consumption to fuel ferroptosis. Specifically, the upregulation of ACSL4 is a rate-limiting step that dictates ferroptosis sensitivity by first activating PUFAs, which are subsequently esterified into membrane phospholipids by enzymes like LPCAT3 (Doll et al., 2017). Therefore, the observed PUFA changes may reflect altered lipid remodeling and peroxidation associated with this pathway, where their consumption is a necessary prerequisite for the generation of peroxidizable lipid substrates within the cell membrane (Kagan et al., 2017). It is this subsequent peroxidation of PUFA-containing phospholipids that ultimately compromises membrane integrity, leading to cell death and the release of pro-inflammatory damage-associated molecular patterns (DAMPs), thereby contributing to the inflammatory phenotype observed in GDM (Tang et al., 2021).

Ferroptosis has recently emerged as a key pathological process in various obstetric complications (Du et al., 2025; Shimazaki et al., 2025). This iron-overload-induced cell death pathway has been implicated in the pathogenesis of conditions such as preeclampsia, gestational diabetes mellitus, recurrent miscarriage, and intrahepatic cholestasis of pregnancy (Katsi et al., 2025). In preeclampsia, for instance, ferroptosis contributes to trophoblast dysfunction and impaired placental angiogenesis, exacerbating maternal cardiovascular risk (Xu et al., 2023). Similarly, in gestational diabetes mellitus, disturbances in iron homeostasis and oxidative stress have been linked to endothelial dysfunction and systemic inflammation, highlighting the role of ferroptosis in disease progression (Katsi et al., 2025). The placenta, as the maternal-fetal interface, is particularly susceptible to ferroptosis due to its high iron content and oxidative environment. Studies have shown that ferroptosis in placental cells leads to compromised trophoblast invasion and maternal-fetal tolerance, which are critical for successful pregnancy outcomes (Khodaei et al., 2025). Furthermore, elevated levels of circulating iron and ferritin, along with increased lipid peroxidation products like malondialdehyde, have been observed in patients with preeclampsia, supporting the involvement of ferroptosis in this condition (Ng et al., 2023). However, our study provides preliminary evidence linking gut microbiota-derived SCFAs to the suppression of placental ferroptosis in GDM. The fact that antibiotic-induced depletion of microbiota and SCFAs phenocopied the placental damage seen in GDM rats supports a contributory role of the gut microbiota in this process.

### 4.3 *In vitro* validation of SCFA protection

Our *in vitro* experiments using an insulin-resistant trophoblast model provided supporting evidence for the protective role of SCFAs. Insulin resistance itself was sufficient to induce ferroptosis, as seen by reduced cell viability, loss of mitochondrial membrane potential, and increases in Fe^2+^, MDA, and ROS. Crucially, co-treatment with a physiological mixture of SCFAs almost completely abrogated these deleterious effects. The protective mechanism was identical to that observed *in vivo*: SCFAs restored GPX4 expression and suppressed the expression of ACSL4 and LPCAT3, thereby preventing the peroxidation of membrane PUFAs. This finding is supported by recent studies showing that butyrate can alleviate ferroptosis in other disease models, such as ulcerative colitis and cardiomyocyte injury, often through the activation of the Nrf2/GPX4 axis (Chen et al., 2024). Our work provides preliminary evidence extending this finding to the realm of placental biology and GDM, though confirmation in primary human trophoblasts from term GDM placentas is needed.

### 4.4 Link to inflammation

The ensuing lipid peroxidation and cellular damage act as potent triggers for inflammation. We documented a significant increase in the serum levels of pro-inflammatory cytokines (TNF-α, IL-6, IL-1β) in GDM rats, which was also replicated in the cell culture supernatant of insulin-resistant trophoblasts. This creates a vicious cycle, as inflammation is both a cause and a consequence of insulin resistance and can further exacerbate cellular stress (Liu et al., 2024). The ability of SCFAs to dampen this inflammatory response may be partly attributable to their effects on ferroptosis, although their well-documented direct anti-inflammatory properties, such as through the inhibition of NF-κB or activation of G-protein-coupled receptors (GPCRs) (Wachamo and Gaultier, 2025; Miao et al., 2024), may also contribute synergistically.

### 4.5 Rationale for using a SCFA mixture

We chose to employ a physiological mixture of acetate, propionate, and butyrate, based on the rationale that these metabolites are simultaneously produced by the gut microbiota in vivo and act synergistically. Using a mixture rather than individual SCFAs more authentically mimics the holistic effects of a healthy gut microbiome on the host. In the physiological environment, SCFAs coexist in specific molar ratios (typically approximately 60:20:20 for acetate, propionate, and butyrate) and jointly regulate host energy metabolism and immune homeostasis (den Besten et al., 2013). Furthermore, different SCFAs possess complementary mechanisms of action; for instance, they activate distinct G protein-coupled receptors (such as FFAR2 and FFAR3) and act as HDAC inhibitors, collectively triggering a broad range of signaling pathways, effects that cannot be fully replicated by any single SCFA (Sun et al., 2017). Therefore, our approach is consistent with several landmark studies in the field that similarly employed SCFA mixtures to investigate their comprehensive physiological effects on metabolism, thereby confirming the validity and relevance of this methodology (Canfora et al., 2017). While dissecting the specific contributions of individual SCFAs represents a valuable future research direction, we believe that using a physiological mixture provides a more comprehensive and relevant model for understanding the overall role of gut microbiota-derived SCFAs in the pathophysiology of gestational diabetes mellitus.

### 4.6 Limitations and future perspectives

Our study has several limitations. First, although ACSL4 and LPCAT3 emerged as key ferroptosis-related molecules in our models, the precise upstream signaling pathways through which SCFAs regulate their expression remain to be elucidated. Second, our animal model used broad-spectrum antibiotics to deplete the microbiota. Although this approach is widely used and effective, it is non-specific, and we cannot fully rule out potential off-target effects of the antibiotics on host metabolism independent of microbiota depletion. Future studies employing germ-free animals, selective bacterial consortium transplants, or additional antibiotic-treated control groups would provide more precise evidence. Third, we utilized a physiological mixture of SCFAs; thus, the relative contribution of each individual SCFA (acetate, propionate, butyrate) to the observed effects warrants further investigation, as they may have distinct and specific roles. Fourth, the largely correlative nature of our findings would be strengthened by genetic gain- or loss-of-function experiments targeting GPX4, ACSL4, or LPCAT3 to establish definitive causality, which represents an important future direction. Fifth, the GC–MS analysis of colonic SCFA levels shown in Figure 3D was performed on only three rats per group, which limits the statistical power and robustness of that specific measurement. Sixth, the lack of fetal biometric data and detailed placental morphometric analysis, such as fetal weight, crown-rump length, and labyrinth zone quantification, limits the comprehensive phenotyping of our model. Future work will benefit from incorporating these structural and developmental parameters. Finally, the *in vitro* experiments were performed in HTR-8/SVneo cells, an immortalized first-trimester trophoblast cell line, which may not fully recapitulate the biological features of placental tissues in later gestation or in clinical GDM. Therefore, the generalizability of these findings to real GDM cases remains limited, and further validation in primary human trophoblasts and placental tissues from patients with GDM will be necessary.

## 5 Conclusion

This study unveils a critical gut microbiota-SCFA-placenta axis in the pathophysiology of GDM. Our findings suggest that SCFA deficiency associated with gut dysbiosis may contribute to placental trophoblast ferroptosis, potentially through modulation of the ACSL4/LPCAT3/GPX4 signaling axis. This not only provides a mechanistic explanation for the placental dysfunction and inflammatory milieu seen in GDM but also opens up new therapeutic avenues. Interventions aimed at restoring a healthy gut microbiota or supplementing with SCFAs and their precursors could represent a promising strategy to mitigate placental injury and improve metabolic health in gestational diabetes.

## Data Availability

The original contributions presented in the study are included in the article/Supplementary material, further inquiries can be directed to the corresponding authors.
